# Atomic-level breakdown of Green–Kubo relations provides new insight into the mechanisms of thermal conduction

**DOI:** 10.1038/s41598-021-84446-9

**Published:** 2021-03-10

**Authors:** Likhith Manjunatha, Hiroshi Takamatsu, James J. Cannon

**Affiliations:** Faculty of Engineering, Department of Mechanical Engineering, Kyushu University, 744 Motooka, Nishi-ku, Fukuoka, 819-0395 Japan

**Keywords:** Fluids, Mechanical engineering

## Abstract

Precise control of thermophysical properties of liquids through tailor-made design of the liquid molecular structure is a goal that, if achieved, could have significant positive impacts on machine design, performance and durability. In this work we show how the breakdown of the Green–Kubo relations down to the atomic level in molecular dynamics simulation can give useful insight into the mechanisms of thermal conduction. Using a group of five small alcohols as a case study, we demonstrate how combining this level of insight with differential-structure analysis reveals the competition for conduction between carbon and hydroxyl group atoms, and show how this competition contributes to the change in thermal conductivity observed in experiment. We hope that this method will become a useful tool in the quest for molecular-structure based thermal design.

## Introduction

Thermal conductivity is a critical property for operation of mechanical and electronic devices. The efficiency of machines and processes are often reliant on the nature of thermal conduction, whether in terms of fast heat extraction from a system or minimising heat loss.

In achieving such heat control, liquids often play an important role, especially with regard to coolants^1^. Moreover, certain industries face reaching limits of mechanical design, such as radiators in automobile applications^2^, which necessitates a focus on better performing heat transfer liquids. Therefore, the ability to precisely control the thermal conductivity of coolant liquids promises to open up customised solutions to various engineering problems.

Although measured macroscopically, the mechanisms of thermal transport in liquids can be traced to their nanoscopic origins, through a combination of phonon transport^3,4^ and diffusion^5^. Molecular Dynamics (MD) simulation, with its ability to probe such scale in detail, is a useful tool to investigate such mechanisms. While description of thermal conduction with MD is typically limited to a classical (non-quantum) framework, it can nevertheless give important insight into the nature and mechanisms of conduction within such a framework of understanding. Thermal conductivity can be determined by molecular simulation through non-equilibrium and equilibrium means; the latter typically employs the Green–Kubo^6,7^ method, which can also be applied in the calculation of diffusion^8^ and viscosity^9^ too. By utilising the fluctuation dissipation and linear response theory^10^, the time correlation of heat flux can be used to derive the conductivity. This avoids certain complexities arising from the large temperature gradients associated with non equilibrium approaches^11,12^. In addition, the Green–Kubo method permits a breakdown of the heat flux and thermal conductivity into constituent parts through analysis of heat flux cross-correlations. Although other methods such as the atomic heat-path method^13^ and the Green–Kubo Modal Analysis method^14^, among others^15–17^, also provide a breakdown of conductivity components, the equilibrium nature and relative simplicity of implementation makes breakdown analysis through the Green–Kubo relations an attractive method^4,5,18–20^.

As a consequence, breakdown through analysis of the cross-correlations of the Green–Kubo method has been utilised to provide useful insight into thermal transport in a variety of situations. For example, English et al.^20^ used the breakdown of correlations to give insight into the contribution to conduction between water molecules, methane molecules and their cross-interactions in methane hydrates. Babaei et al.^5^ used such breakdown to show how thermal transport through molecular interaction rather than molecular diffusion is the dominant mode of transport in nano-fluids. Such breakdown has also been shown to give useful insight into contributions to conductivity from short and long wavelength phonons^4^.

While such studies have provided useful insight into the nature of thermal conductivity, an analysis of the very influential atomic-level pairwise interaction contributions has, to the best of our knowledge, not yet been performed. This information is important from a molecular design perspective, since it potentially enables prediction of how changes in atomic composition and structure cause corresponding changes in inter-atomic conductivity and hence overall conductivity. In order to realise this, we have investigated atomic-level correlations obtained through breakdown of the Green–Kubo correlations to give useful information about atomic pairwise contribution to conductivity. To demonstrate the technique’s utility, we have used this technique to give insight into conductivity of a set of small alcohols: ethanol, propanol, ethylene glycol (EG), propylene glycol (PG) and 1,3 propanediol (13P). These are chosen for their structural similarity, usefulness in common applications, and available data in existing literature.

**Figure 1:**
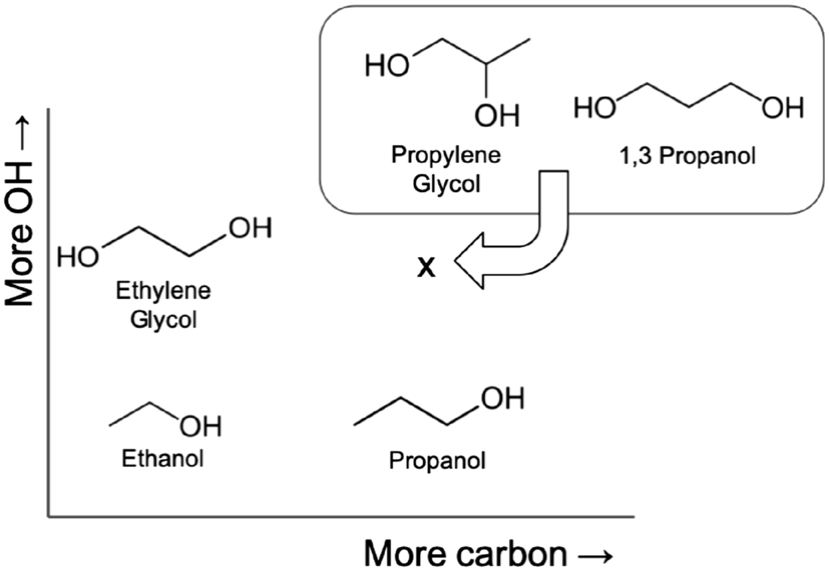
The 5 alcohol molecules studied in this work and the relation of their structures.

**Figure 2:**
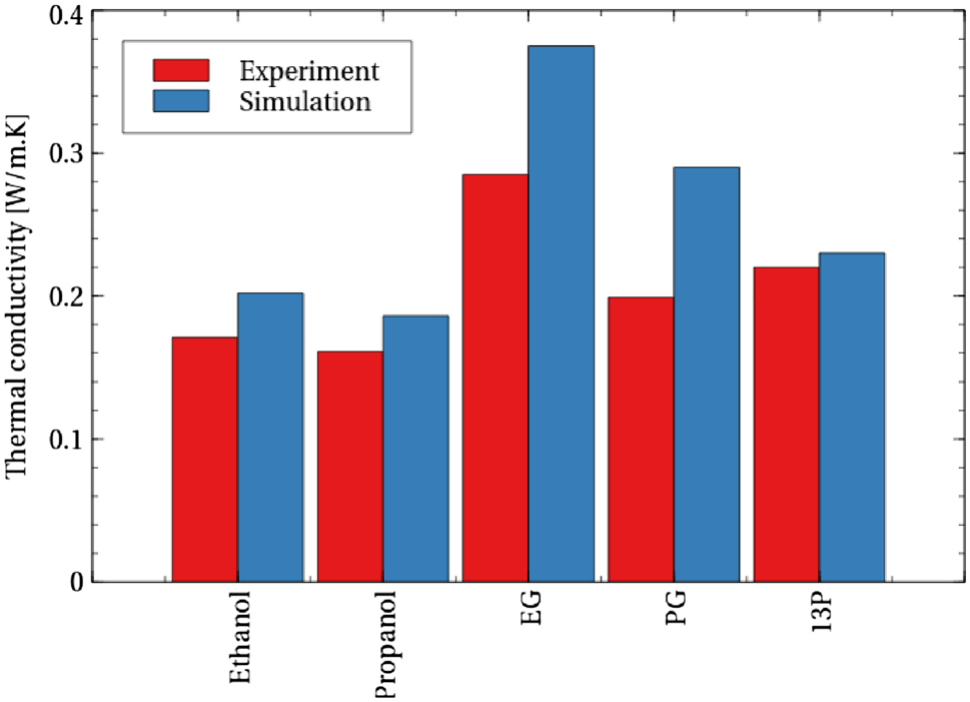
A comparison of the overall thermal conductivity at 300 K between the simulation models used in this work and experiment^21–23^. As is typical in molecular simulation, thermal conductivity is generally over-estimated, and with the exception of PG-13P, relative values are maintained.

Although adding one carbon atom to the carbon chain of ethanol to make propanol (Fig. 1) is known to result in little change in thermal conductivity (Fig. 2), the result is very different if a hydroxyl (–OH) group is added instead to make EG. Having long been used as a working alcohol in antifreeze technologies, simulation studies have shown that the hydroxyl groups of EG contribute to its large thermal conductivity^16^. Adding both a carbon and hydroxyl group to ethanol (that is, to add a hydroxyl group to an internal carbon of propanol to make PG or to the end to make 13P) is also known to increase thermal conductivity compared to ethanol^23^, although not to the extent of EG. Until now, however, the atomic mechanisms responsible for such variation have remained unclear.

In this study, through differential structure analysis and corresponding atomic-level breakdown of contributions to conductivity, it is shown that enhanced understanding of how changes in molecular structure cause nano-scale changes in pairwise conduction can be obtained. In this work, the most important modes of heat transfer are highlighted and the thermal conductivity components from pairwise atomic interactions are explicated to understand the macroscopic changes in conductivity of different molecules. The outline of this manuscript is as follows. The next section details the simulation set-up, and the thermal conductivity calculation procedure is presented. This is followed by a progressive breakdown of overall thermal conductivity, eventually down to the atomic-level pairwise term. Each level of breakdown is followed by detailed interpretation of the effect of structural change on these thermal conductivity components. Finally, the wider implications of this technique are discussed.

## Simulation details

Thermal conductivity values are calculated using equilibrium molecular dynamics with the Green–Kubo method. More specifically, the heat flux vectors and their correlations are calculated throughout the simulations, and an integration of the heat flux auto-correlation function over time is used to calculate the thermal conductivity (Eq. 1).

1 $$\begin{aligned} \lambda = \frac{V}{k_B T^2} \int _0^\infty \langle J(t).J(0) \rangle dt \end{aligned}$$

Here, *J*(*t*) is the heat flux vector at time *t*; *V* and *T* are the volume and temperature of the system, respectively, while $k_B$ is the Boltzmann constant. The total heat flux is subjected to three levels of breakdown. Firstly, it is isolated into a convective term $J_c$ arising primarily from diffusion of particles, and a virial term $J_v$ primarily from atomic interactions (Eq. 2 to 4). The convective term (Eq. 3) depends on the per-atom energy $e_i$ and velocity $v_i$ of each atom *i*, while the virial term (Eq. 4) depends on the force $F_{ij}$ between atoms *i* and *j*, their velocities $v_i$ and $v_j$ and their separation $r_{ij}$.

2 $$\begin{aligned} J(t) =&J_c(t) + J_v(t) \end{aligned}$$

3 $$\begin{aligned} J_c =&\frac{1}{V} \left[ \sum _i e_i v_i \right] \end{aligned}$$

4 $$\begin{aligned} J_v =&\frac{1}{2V} \left[ \sum _{i<j} (F_{ij} . (v_i + v_j)) r_{ij} \right] \end{aligned}$$

This results in auto-correlation and cross-correlation contributions to overall thermal conductivity (Eq. 5 and 6).

5 $$\begin{aligned} \lambda&= \frac{V}{k_B T^2} \int _0^\infty \langle (J_c(t) + J_v(t)).(J_c(0) + J_v(0)) \rangle dt \end{aligned}$$

6 $$\begin{aligned}&= {\lambda }_{cc} + {\lambda }_{vv} + {\lambda }_{cv} + {\lambda }_{vc} \end{aligned}$$

$\lambda _{cc}$ corresponds to the auto-correlation between the convective terms (Eq. 7), while $\lambda _{vv}$ corresponds to the same quantity for the virial terms. Cross-correlations are similarly given by ${\lambda }_{cv}$ and ${\lambda }_{vc}$.

7 $$\begin{aligned} \lambda _{cc} = \frac{V}{k_B T^2} \int _0^\infty \langle J_c(t).J_c(0) \rangle dt \end{aligned}$$

Substituting this into the Green–Kubo relation gives three conductivity components: two from auto-correlations of convective and virial terms each and a cross-correlation term. The virial term $\lambda _{vv}$ is further decomposed in terms of bonded and non-bonded conduction as well as intra- and inter-molecular conduction. This is achieved by breaking the flux $J_v$ (Eq. 8) into components and isolating those arising from external pair interactions $J_{ep}$, internal pair interactions $J_{ip}$, long-range coulomb interaction $J_{lc}$ and flux arising from bonded conduction through the molecule $J_b$. This is possible because molecular dynamics simulation forcefields, including the OPLS-AA forcefield used here, define the molecules and their interactions through such distinct interactions.

8 $$\begin{aligned} J_v = \overbrace{J_{ep} + J_{ip} + J_{lc}}^{\text {non-bonded}} + \overbrace{J_{b}}^{\text {bonded}} \end{aligned}$$

Internal ($J_{ip}$) and external ($J_{ep}$) pairwise flux can be further divided into contributions from heat-transfer interactions between constituent atoms: in this case carbon (C), oxygen (O) and hydrogen (H).

9 $$\begin{aligned} J_p = \sum {J_{XY}}, \quad X, Y = C, O, H \end{aligned}$$

Ultimately, this allows us to break the non-bonded pair interaction contribution to the original flux correlation of Eq. 1 down to the correlations of the heat flux arising from specific atomic interaction pairs. For example, $\lambda _{OH{-}OH}$ from the $\langle J_{OH} . J_{OH} \rangle$ auto-correlation is the contribution to thermal conductivity from heat flux due to oxygen–hydrogen interactions. A contribution from $\lambda _{OH{-}CO}$ arising through $\langle J_{OH} . J_{CO} \rangle$ cross-correlation likewise corresponds to contribution to thermal conductivity from heat flux due to oxygen–hydrogen interactions correlated with carbon–oxygen interactions.

Non-trivial cross-correlations are a good indicator of the nature of influence between the two kinds of interactions: a positive, negative or zero cross-correlation value indicates a collaborative, competitive or independent relationship respectively between two interaction types. All this information gives unprecedented insight into the nano-scale mechanisms and understanding about the nature of thermal conductivity in liquids.

The simulations were conducted using LAMMPS^24^. The stock LAMMPS code provides the ability to break heat flux components down to interaction type such as non-bonded (short-range), non-bonded (long-range), bond, angle and so on. These contributions were combined appropriately to give meaningful quantities, and the code was customised further to provide heat flux breakdown to the atomic level and enable discrimination between internal and external pairwise conduction of molecules. The OPLS-AA force field^25^ was used to describe the molecular interactions of the alcohols under study and long-range Coulomb interactions were calculated using the particle–particle–particle method (pppm)^26^ with a cutoff distance of 10 Å; this distance chosen for maximum computational speed whilst retaining sufficient accuracy. Density was underestimated from the experimental values^27–30^ by between 0.8% (propylene glycol) and 4.6% (propanol). While 10 Å is not unprecedented^31^, a 12 Å cutoff is perhaps more common for studies of this nature^15,16,20,32^, however in tests under the conditions used in this study with ethylene glycol we found the difference in density, thermal conductivity and its components to be negligible under these two cutoff distances. Length of bonds involving one hydrogen bond, and angles involving two hydrogen bonds were fixed using the SHAKE algorithm^33^. Any influence on the thermal conductivity by SHAKE is incorporated into the “bonded” contribution to conductivity. Since the overall thermal conductivity is well represented by the model, we assume that any influence on thermal conductivity by SHAKE is properly accounted for.

The initial dimensions of all systems were that of a cubic box of side length 40 Å with periodic boundary conditions applied with minimum image convention in all three directions. The number of molecules was constrained by the liquid’s experimental density and the initial box size. All simulations were run with a time step of 1 fs. The system was minimised for a short 100 ps in the NVT ensemble and equilibrated for a further 20 ns at constant pressure (1 atm) and temperature (300K). This NVT ensemble is achieved using a Nosé–Hoover^34,35^ thermostat with a 0.1 ps coupling time. The production stage was preceded by a short transition stage to ensure no residual influence from the thermostat before sampling. Sampling in each case occurred for 2 ns; long enough to ensure proper convergence and sampling for every system studied here.

The running integral of the Green–Kubo calculation for each molecule was typically averaged over 100 simulations to get a representative sampling of the phase space and improve the statistics of the calculations. Since short-time correlations are averaged over more samples than long-time correlations, the integration curve gets noisier as the correlation time gets longer^36^. Moreover, since the correlation functions never truly converge to zero, but instead fluctuate around it, the running integral accumulates error as correlation time gets longer, resulting in larger deviations as time progresses. Since the running integral therefore does not plateau to a constant value, the theoretical evaluation of thermal conductivity at infinite correlation time is not possible. Instead, we have adopted a heuristic method of curve-fitting similar to that of Chen et. al.^37^ to overcome this problem utilising multiple measurements of correlation at short correlation times.

The curve-fitting method involves calculating the standard error of the mean (SEM) of all runs at all correlation times using Eq. (10), and using this to weight the curve-fit of Eq. (11), where $A_1$, $A_2$ and $Y_0$, $\tau _1$ and $\tau _2$ are empirical fitting parameters, with the latter two representing time decay constants^37^. Since the SEM increases progressively with time, the points at later time are given less weighting when fitting the curve.

10 $$\begin{aligned} \sigma (x)= & {} \sqrt{\frac{1}{N-1} \sum _{i=1}^N(\lambda (x)_i - \langle \lambda (x)\rangle )^2} \end{aligned}$$

11 $$\begin{aligned} \lambda= & {} A_1\exp ^{\frac{-t}{\tau _1}} + A_2\exp ^{\frac{-t}{\tau _2}} + Y_0 \end{aligned}$$

To ensure that all components add up accurately to the total conductivity, the following algorithm is implemented. Firstly, considering the flux correlation for total thermal conductivity, all intervals [*a*, *b*] in which the running integral of the correlation lay within 3% of the plateau value obtained by the curve fit are noted. Each interval is evaluated based on a score *s* prioritising greater interval size and earlier starting time *a* (Eq. 12). Greater interval size suggests greater stability of the correlation while intervals with earlier starting time have smaller errors than ones at a later time.

12 $$\begin{aligned} s = \frac{\text {length of the interval}}{\text {starting point of interval in time}} = \frac{b-a}{a} \end{aligned}$$

**Figure 3:**
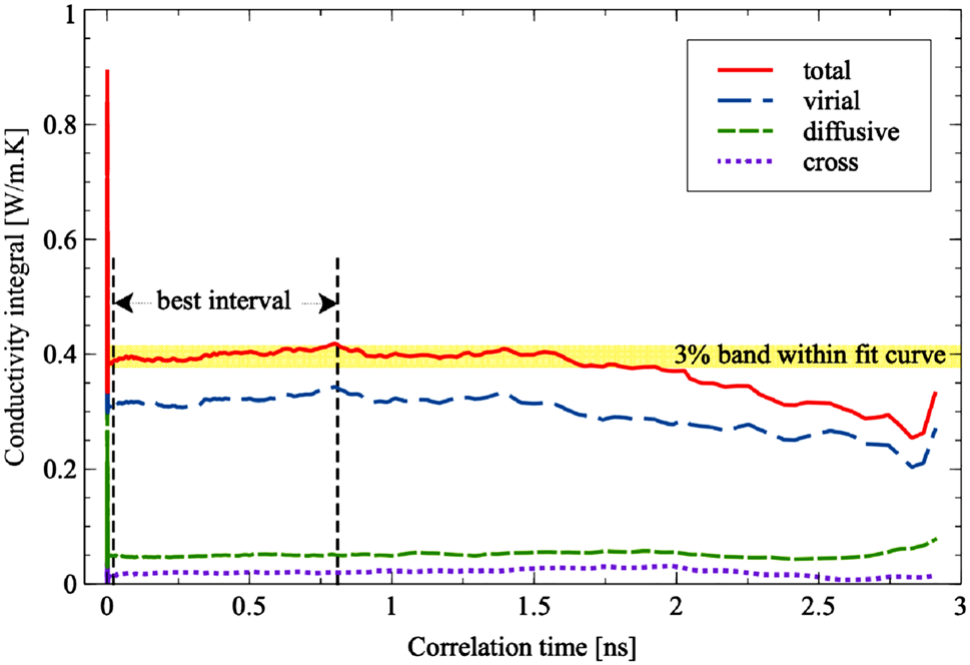
Heat-flux autocorrelation integral curve for virial, diffusive and the total conductivity. The 3% band around the plateau value is highlighted, and the corresponding best interval (Eq. 12) is shown. The average thermal conductivity is then measured over this best interval, and all contributions to the conductivity here use the same interval, ensuring that the sum of the components (in this case, virial and diffusive conduction) add up to the total.

Once the interval with highest score *s* is obtained, the total thermal conductivity is obtained by taking the average of the running integral within that interval. When breaking down the total thermal conductivity into sub-components, the contribution of each component is calculated by averaging each respective running integral over the same interval. This algorithm permits rapid and accurate computational evaluation of the many contributions to the thermal conductivity while ensuring that the sum remains equal to the total conductivity. Figure 3 shows an example highlighting the 3% band and subsequent best interval as determined from the score *s*.

It has recently been shown by Boone et. al.^38^ that long molecules can have their internal thermal conductivity under-estimated when using the Green–Kubo method to calculate heat flux in LAMMPS. This is due to incorrect calculation of the angle (3-body) and dihedral (4-body) contributions to heat flux. In the current study, the longest molecule considered only has a carbon chain length of 3 atoms and hence the influence on the results here is expected to be negligible.

## Results and discussion

### Virial and convective conduction

**Figure 4:**
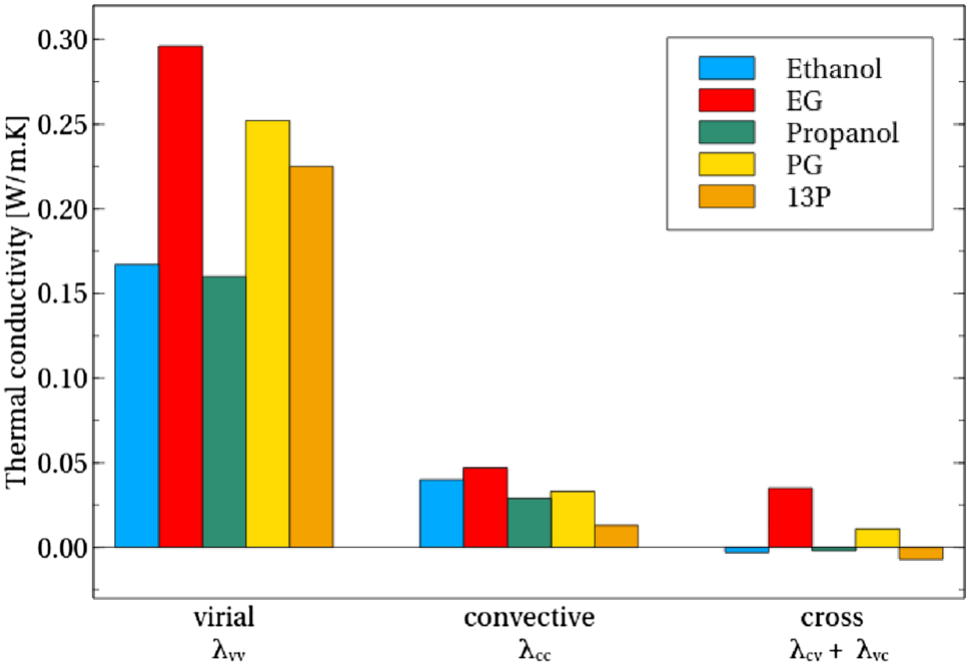
The virial contribution to conductivity not only contributes the most to thermal conductivity for each molecule, but is also the driving contribution behind much variation between the molecules.

Thermal transport through atomic-level interaction between atoms is represented by the virial contribution, $\lambda _{vv}$, to thermal transport (Eq. (6)). Before examining this contribution in detail, it is important to clarify the importance of the virial contribution to the overall thermal conductivity. Thus, the thermal conductivity breakdown into virial, convective and their cross-correlation terms is calculated for the molecules ethanol, propanol, EG, PG and 13P (Fig. 4). The virial contribution is clearly observed to be the main contribution to the thermal conductivity. Not only is the relative magnitude of the contribution large but it appears to be the primary driver in the variation of conductivity between the molecules. The overall importance of the virial term for thermal conduction in liquids is in common with previous literature^5,39,40^. Meanwhile, the convective and cross terms show relatively small contribution and variance with structural differences.

The virial term is a collection of bonded (angle, torsion, bond, etc) and non-bonded (pairwise coulomb and van der Waals) interactions which are easily affected by changing the structure of molecules. Increasing the chain length of alcohol, for example, increases intramolecular heat conduction from angle and torsion terms but reduces intermolecular heat transfer due to hydroxyl group(s)^13,15^, while multiple hydroxyl group substitution affects heat transfer through competing inter- and intra-molecular hydrogen bond interactions^39^. The virial term therefore holds important information on heat conduction dynamics and demands a deeper investigation.

### Bonded and non-bonded conduction

**Figure 5:**
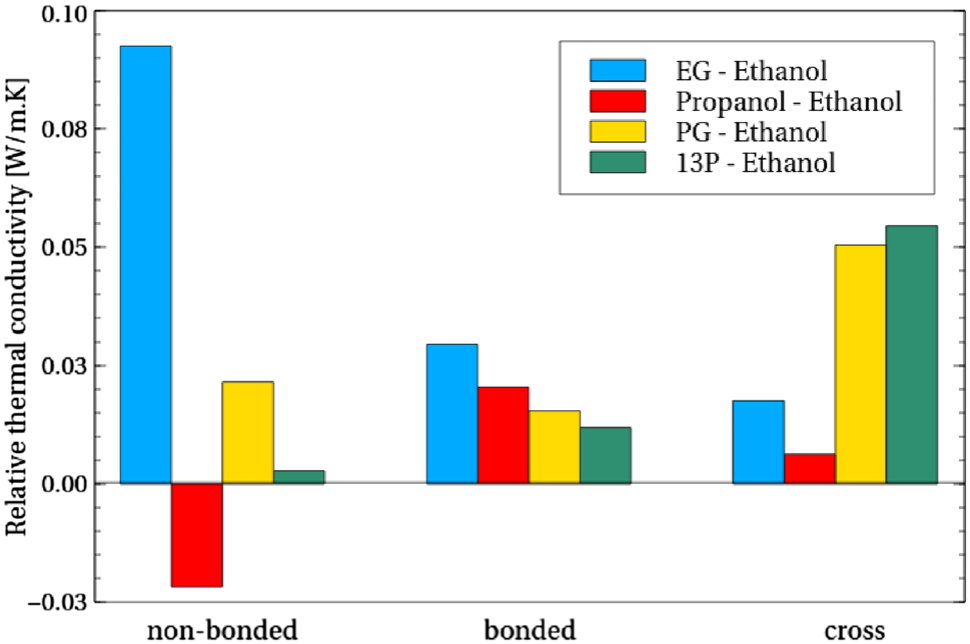
The change in conductivity contribution when adding atoms to ethanol. All values are shown relative to that for ethanol. A positive value means that the other molecule has a greater contribution than ethanol for that type of conduction.

To obtain a deeper understanding, the virial term contribution to conductivity is further split into constituent contributions from bonded, non-bonded and cross correlation terms. In order to make the relative contributions of structural changes clear, ethanol is chosen as a base alcohol and comparison of contributions is made relative to this (Fig. 5).

It is interesting to note that non-bonded conduction appears to be the greatest factor in deciding how adding atoms to ethanol effects the conduction. While there is some variation in how contribution from bonded and cross-correlations change, non-bonded contributions vary most significantly among the four molecules, from strongly positive increase (EG) to a negative influence (propanol) and in one case (13P) largely no influence at all. In the case of EG, it is known that there is significant hydrogen bonding^41^ and such bonding is known to promote strong thermal conductivity^42^, so it is likely that addition of the hydroxyl group results in a greater number of hydrogen bonds and hence a larger thermal conductivity.

The picture appears to be more nuanced, however, than simply modifying the number of hydroxyl groups to modify the non-bonded conductivity. Placing a carbon atom to extend the carbon chain of ethanol (creating propanol) causes a reduction in the non-bonded contribution to thermal conductivity. Adding this carbon atom to ethanol and then placing a hydroxyl group on the end of the carbon chain to form 13P also results in two hydroxyl groups, just as EG has, but the non-bonded contribution to conduction barely changes. Placing that hydroxyl group inside the carbon chain to recover the original distance between the hydroxyl groups (resulting in PG) does however promote non-bonded conduction, suggesting this separation is important for promotion of such non-bonded conduction, although the increase is not to the extent observed for EG.

**Figure 6:**
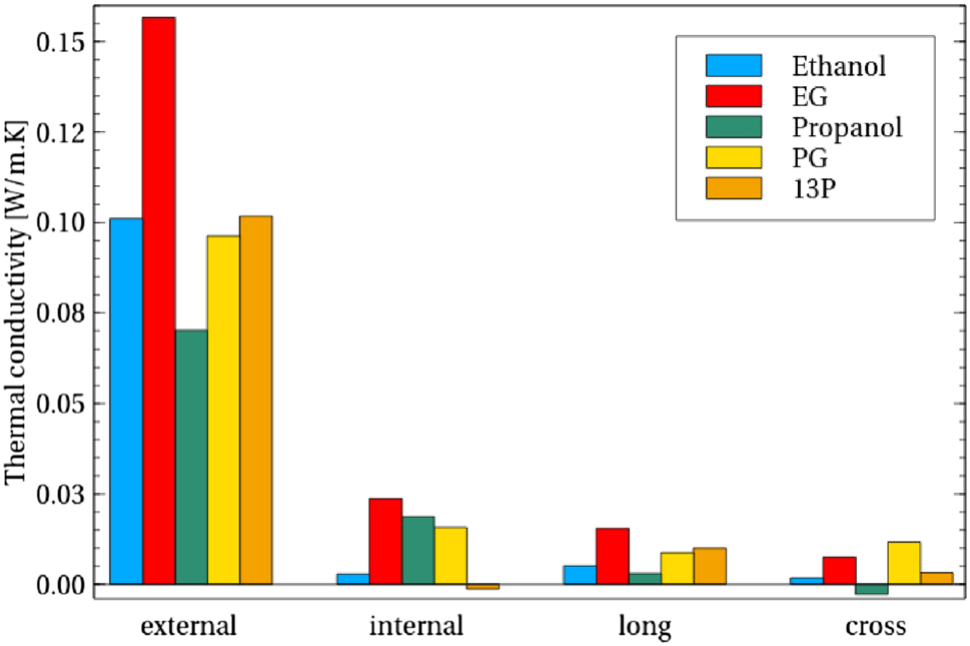
Breakdown of non-bonded contribution to conductivity by short-range external and internal non-bonded interaction, as well as long-range interactions and their cross-contributions.

Interestingly, focusing on short-range non-bonded conduction, our calculations suggest that PG and 13P show similar external (inter-molecular) conduction as ethanol (Fig. 6), but the internal non-bonded conduction of PG contributes to its overall higher conductivity observed in experiments, indicating that shorter separation between hydroxyl groups promotes internal non-bonded thermal conductivity possibly through internal hydrogen bonding.

Bonded conduction meanwhile increases for any addition to ethanol (Fig. 5), although the extent of the increase is again dependent on the nature of the addition and not simply that the molecule is longer. Indeed, 13P, despite being longer than EG, has lower bonded conductivity. Meanwhile, conduction from cross-correlation between bonded and non-bonded contributions is particularly large for PG and 13P, showing that the influence of these cross-correlations is more significant for these larger molecules.

### Atomic-level understanding of conduction

Although we understand the importance of the virial contribution to thermal conductivity and the role of bonded and non-bonded contributions from the aforementioned results, the nature of hydroxyl group conduction mentioned earlier warrants further investigation, since the number and positioning of such groups appears to play a critical role in the mechanisms of thermal conductivity. To do this, an atomic-level investigation of conductivity is necessary. In particular, the nature of such conductivity and the hydroxyl group’s interplay with other atoms present must be clarified.

**Figure 7:**
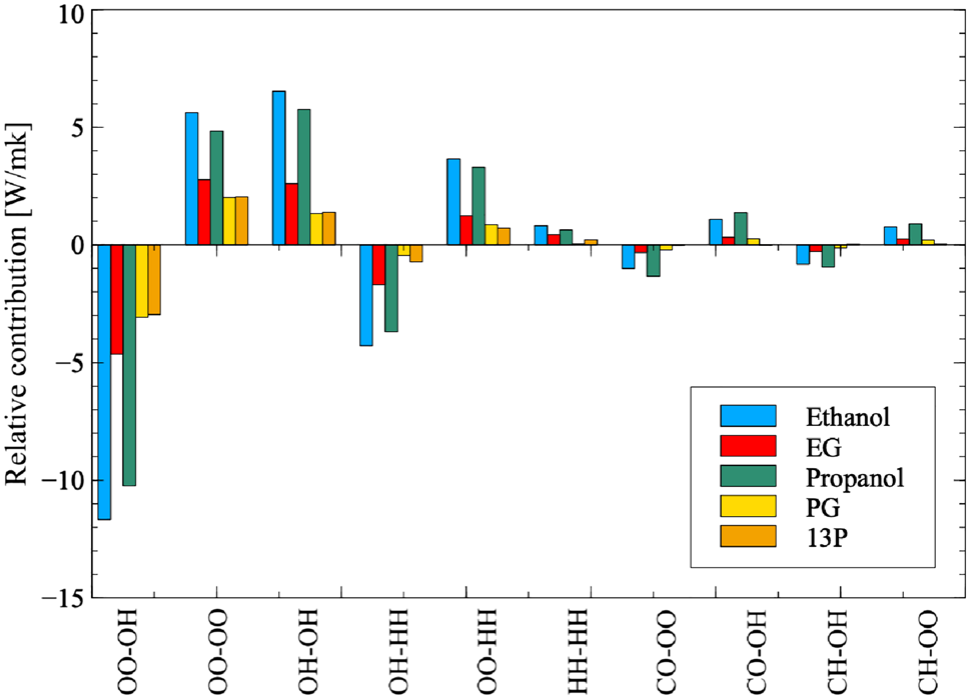
The relative contribution of the top 10 atomic-correlation contributors (out of a total of 55) to the thermal conductivity of each molecule (Eq. 13). Contributors are ordered by magnitude, with positive and negative values preserved in the graph. For each molecule, the sum of all 55 relative contributions will add to one. Correlations between oxygen (O), hydrogen attached to oxygen (H) and carbon (C) atoms are represented on the x-axis. Hydrogen atoms attached to carbon atoms do not feature in the top 10 contributors and are therefore not shown. For example, *CO–OH* represents the contribution of correlation of flux between carbon and oxygen atoms with flux between oxygen and hydrogen atoms, while *OO–OO* represents the auto-correlation of flux between oxygen atoms only.

Firstly, in order to identify the most important atomic interactions for thermal conductivity, all atomic interactions are ordered by their magnitude of contribution to the thermal conductivity of each molecule relative to the total inter-molecular non-bonded thermal conductivity (Eq. 13).

13 $$\begin{aligned} \lambda _{contrib} = \lambda _{WX-YZ} / \lambda _{inter,nb} \end{aligned}$$

where $\lambda _{WX-YZ}$ describes the thermal conductivity due to the correlation between W–X atom interactions and Y–Z atom interactions and $\lambda _{inter,nb}$ is the total inter-molecular non-bonded thermal conductivity. In doing this, it is observed that the 10 largest atomic contributions are common among all molecules studied here (Fig. 7).

It is evident from Fig. 7 that the main contributors to thermal conductivity are overwhelmingly from correlations involving oxygen and hydrogen atoms. In fact, all six correlations that occur purely due to hydroxyl group interactions also happen to occupy spots among the top ten contributors to conductivity for all molecules under study. This supports the earlier suggestion that the non-bonded conduction is primarily occurring through hydrogen bonding which is a product of interactions of these two types of atoms.

**Figure 8:**
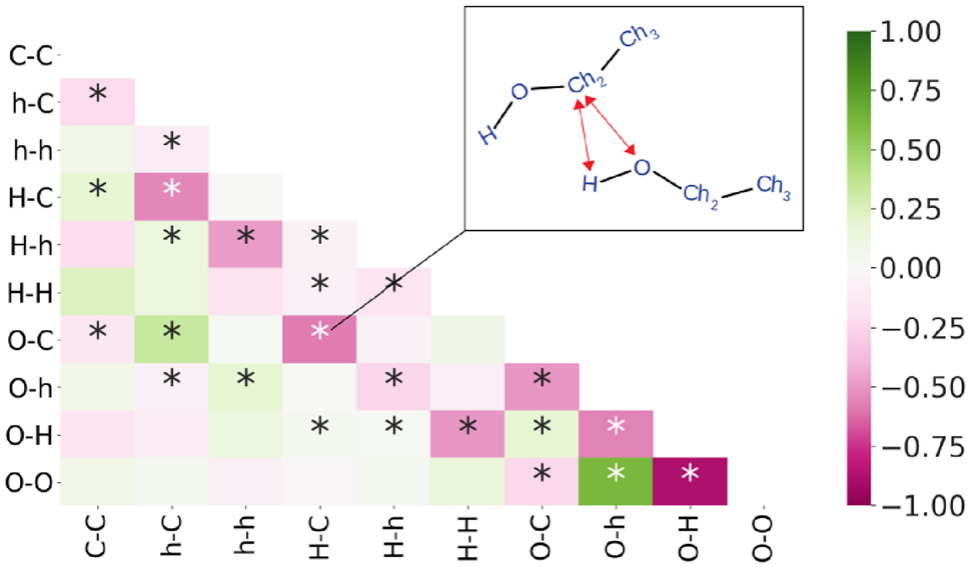
Pearson correlation coefficient map of heat flux correlations between pairs of atoms for ethanol. Correlations that share a single atom type are marked with a star. Oxygen (*O*), carbon (*C*), hydrogen attached to oxygen (*H*) and hydrogen attached to carbon (*h*) are all shown. The inset figure highlights one example of correlations; here between H–C and O–C atoms on two molecules. Note that there may be multiple ways to form each correlation depending on the number of occurrences of the atom type on the molecule.

What is striking in this analysis, and occurred to a lesser extent in earlier analysis, is the presence of negative correlations in the heat flux. The sum of the correlations gives the thermal conductivity which is always positive, but in order to gain an insight into the meaning of these component negative correlations it is interesting to consider the correlations of the heat fluxes themselves (Fig. 8).

By mapping the correlation coefficients of the instantaneous heat fluxes, anti-correlation between fluxes that share a single common atom becomes clear, showing an average Pearson correlation coefficient (PCC) of − 0.15, in contrast to pairs of atoms that do not share a common atom which have an average PCC roughly an order of magnitude smaller, much closer to zero (0.02). The negative value suggests that there is “competition” for the flux between atom pairs that share a common atom, particularly for atoms related to hydrogen bonding. As is demonstrated by the PCC value of − 0.8 for the interaction pairs O–O and O–H, a configuration that promotes the flow of heat between oxygen atoms corresponds to “anti-” flow of heat between oxygen and hydrogen, suppressing the thermal conductivity, and vice versa. By contrast, the flux between oxygen atoms happens largely independently to that between hydrogen atoms, since there are no shared atoms, hence the value close to zero in the PCC map. While there are some exceptions to the rule, the average influence of having a common atom is substantially more negative than without, suggesting that conduction through one type of atom is usually dominated by conduction with another atom at the expense of other atoms, especially in the case of hydroxyl group atoms.

This gives insight into the positive and negative nature of correlations observed in Fig. 7. Self-correlations of oxygen–oxygen interaction and oxygen–hydrogen interaction result in a positive contribution to thermal conductivity, as does the cross correlation between oxygen–oxygen flux and hydrogen–hydrogen flux. Meanwhile, correlations that are “competing” for an atom; oxygen and hydrogen competing for correlation with another oxygen atom O–O correlation and O–H correlation (OO–OH), as well as H–H with O–H (HH–OH); result in a negative contribution to conductivity.

It is striking to note that ethanol by far has the largest magnitudes for thermal conductivity; both positive and negative. These ultimately cancel out however, and EG ultimately has the largest thermal conductivity. Generally, the smaller the molecule, the larger the magnitude of the correlations, suggesting that thermal conductivity is more sensitive to structural modification for smaller molecules.

**Figure 9:**
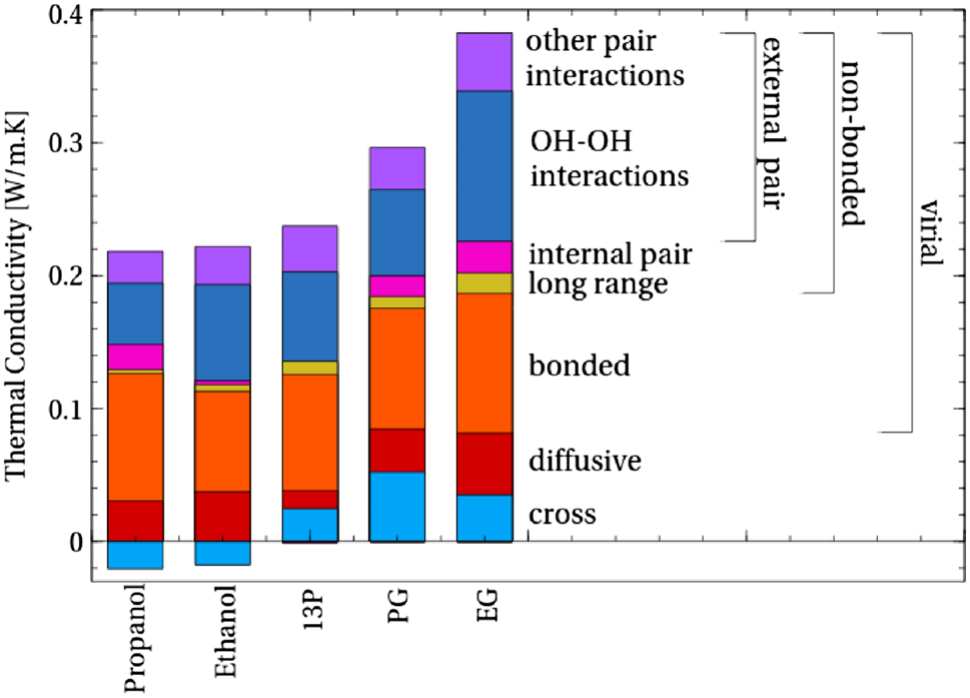
A summary of all the components of thermal transport within the molecules studied. The virial contribution to conductivity not only contributes the most to thermal conductivity for each molecule, but is also the driving contribution behind much variation between the molecules. Of this, the external (inter-molecular) hydroxyl group (OH–OH) contribution to conductivity is significant.

The contribution of these atomic level contributions to thermal conductivity are shown in context of the wider picture of all contributions to thermal conductivity in Fig. 9.

### Effect of structural changes on thermal conductivity

With this new level of understanding of thermal conduction on the atomic level, we can begin to consider the mechanisms of heat transfer.

**Figure 10:**
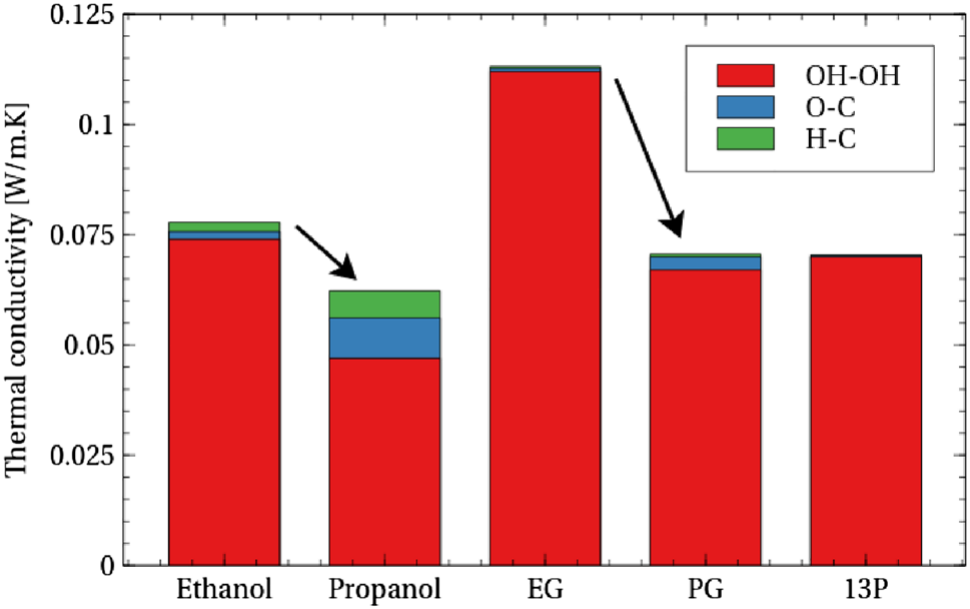
Addition of carbon to Ethanol and EG to form Propanol and PG respectively results in decreased external conduction between hydroxyl groups (OH–OH) but increased internal oxygen–carbon (O–C) and hydrogen (attached to oxygen)–carbon (H–C) conduction.

The nature of carbon disruption of conduction between hydroxyl groups can be quantitatively analysed. By comparing Ethanol to Propanol and EG to PG, the effect of adding a primary carbon atom on the intermolecular conduction between hydroxyl groups (“OH” in Fig. 10) is striking, with a significant decrease observed in both cases. In contrast however, the C–O and C–H autocorrelation contributions to intramolecular conductivity can be seen to increase. The conduction between hydroxyl groups is clearly more sensitive however, and this increase is not enough to offset the loss of conductivity between hydroxyl groups.

While there is a net decrease in thermal conductivity in both cases, the role of the added carbon atom appears to be different. In the case of adding a carbon atom to Ethanol to make Propanol, a significant amount of conduction between hydroxyl groups is replaced by conduction between oxygen and hydrogen with carbon. Thus the addition of the carbon atom creates alternative hydroxyl-carbon pathways for thermal transport. By contrast, in moving from EG to PG, the increase in conduction with the carbon atom is much smaller. This suggests that the addition of the carbon atom is disrupting the conduction between hydroxyl groups without creating a significant alternative conduction pathway. Therefore, although in both cases the carbon atom is acting to disrupt conduction between hydroxyl groups, the extent that it can replace lost conductivity is clearly different, and the details of such difference would not be identifiable without the atomic-level breakdown of the conductivity employed here.

Another way to add a carbon atom to EG is to add the carbon inside the chain as a secondary carbon to form 13P. This actually reduces the C–O and C–H autocorrelation contributions to thermal conductivity slightly, suggesting that the fact that the added carbon atom is primary is important for realising conduction with hydroxyl group atoms.

In this study we have focussed on five small alcohols. To generalise these results to a larger range of molecules, it is necessary to consider what molecular structures experience this competition between hydroxyl groups and carbon atoms for conduction with other hydroxyl groups. Since the net effect of adding the carbon atom is to reduce thermal conductivity, it seems reasonable to expect that any decrease in conductivity should be closely related to the proximity of the added carbon to a hydroxyl group, since this increases the probability of interaction between the two.

The variation of conductivity of linear alcohols with chain length appears to support such intuition. As the number of carbon atoms in a molecule increases, conductivity initially drops for smaller chain alcohols, reaches a minimum, and rises negligibly for longer chains^15,43,44^. This is in line with our results, since this suggests that conduction of short chains and long chains are characterised by different influencing factors. The influence of intramolecular C–O and C–H interaction is significant for short chain alcohols due to the proximity of the added primary carbon to the hydroxyl group. But as the chain gets longer, the influence of C–O and C–H interactions due to the added carbon becomes negligible and change in conductivity is instead dominated by internal conduction, resulting in the slight increase in conduction in longer chains.

## Conclusion

We have demonstrated how breakdown of the Green–Kubo correlations down to atomic-level correlations in molecular dynamics simulation can give valuable information about the mechanisms of thermal conductivity.

As an example of this technique’s utility, we have combined this breakdown analysis with differential structure analysis to compare pure liquids of five small alcohols and understand why changes in conductivity occur when adding or subtracting atoms. While experimental observations of thermal conductivity may allow indirect inference of the importance of the presence of certain atoms or interactions, this method has been shown to give definitive information about contribution from inter-atomic interactions and their competition in determining overall thermal conductivity. For the case of the small alcohols studied here, we have detailed how competition for interaction with hydroxyl group atoms plays a major role in deciding the overall thermal conductivity. For generalisation to other molecules, we have demonstrated how the effects of such competition are likely to be most acute in cases where the addition of a primary carbon atom is close to a hydroxyl group.

While this technique provides valuable information, the biggest challenge is perhaps the computational cost. The Green–Kubo method is well known to be noisy, and breaking down the correlations into atomic-level sub-correlations requires yet further independent repeated calculations to reduce the noise to acceptable levels. Nevertheless, while this technique may initially find utility with small molecules, as computational power continues to increase we hope this technique will play an ever-greater role in understanding mechanisms of thermal conductivity and aid in molecular design of liquids and materials with precisely designed thermophysical properties.
